# GLP-1R as a potential link between diabetes and Alzheimer’s disease

**DOI:** 10.3389/fnagi.2025.1601602

**Published:** 2025-07-24

**Authors:** Shujun Li, Nanqu Huang, Mei Wang, Wendi Huang, Jingshan Shi, Yong Luo, Juan Huang

**Affiliations:** ^1^Key Laboratory of Basic Pharmacology and Joint International Research Laboratory of Ethnomedicine of Ministry of Education, Zunyi Medical University, Zunyi, Guizhou, China; ^2^Department of Geriatrics, National Drug Clinical Trial Institution, Third Affiliated Hospital of Zunyi Medical University (The First People’s Hospital of Zunyi), Zunyi, Guizhou, China; ^3^Department of Neurology, National Drug Clinical Trial Institution, Third Affiliated Hospital of Zunyi Medical University (The First People’s Hospital of Zunyi), Zunyi, Guizhou, China; ^4^Chinese Pharmacological Society-Guizhou Province Joint Laboratory for Pharmacology, Zunyi, Guizhou, China

**Keywords:** glucagon-like peptide-1 receptor, Alzheimer’s disease, diabetes mellitus, insulin resistance, neuroinflammation

## Abstract

There is growing interest in the relationship between Alzheimer’s disease (AD) and diabetes mellitus (DM), and the glucagon-like peptide-1 receptor (GLP-1R) may be an important link between these two diseases. The role of GLP-1R in DM is principally to regulate glycemic control by stimulating insulin secretion, inhibiting glucagon secretion, and improving insulin signaling, thereby reducing blood glucose levels. In AD, GLP-1R attenuates the pathological features of AD through mechanisms such as anti-inflammatory effects, the reduction in amyloid-beta (Aβ) deposition, the promotion of Aβ clearance, and improvements in insulin signaling. Notably, AD and DM share numerous pathophysiological mechanisms, most notably the disruption of insulin signaling pathways in the brain. These findings further underscore the notion that GLP-1R plays pivotal roles in both diseases. Taken together, these findings lead us to conclude that GLP-1R not only plays an important role in the treatment of DM and AD but also may serve as a bridge between these two diseases. Future research should focus on elucidating the detailed molecular mechanisms underlying the actions of GLP-1R in both diseases and exploring the development of GLP-1R agonists with dual therapeutic benefits for AD and DM. This could pave the way for innovative integrated treatment strategies to improve outcomes for patients affected by these intertwined conditions.

## 1 Introduction

Alzheimer’s disease (AD) is a progressive neurodegenerative condition characterized by memory loss and cognitive dysfunction (Scheltens et al., 2021). According to statistics, there are currently approximately 50 million people living with AD worldwide, and this number is expected to increase to 152 million by 2050 as the population ages, making it a major challenge for global public health (Dissanayaka et al., 2024). Despite extensive research and clinical trials conducted on the underlying mechanisms, the etiology and pathogenesis of AD remain incompletely understood.

The glucagon-like peptide-1 receptor (GLP-1R) is a key target for diabetes mellitus (DM) treatment, and GLP-1R agonists are pharmaceutical compounds employed in the treatment of DM. However, recent studies have revealed the potential for these compounds to also impact the pathological process of AD. These impacts include anti-inflammatory effects, reduced amyloid-beta (Aβ) deposition, reduced tau protein hyperphosphorylation, and improved insulin signaling (Calsolaro and Edison, 2016; Kang et al., 2023). Therefore, the utilization of GLP-1R medications in the treatment of AD may represent a promising approach. Notably, AD and DM share multiple pathophysiological mechanisms, and in particular, both AD and type 2 DM (T2DM) disrupt insulin signaling pathways in the brain (Barone et al., 2021). In addition, 60–70% of patients with T2DM suffer from cognitive impairment (Biessels and Despa, 2018). Thus, GLP-1R may be a potential link between DM and AD, and we look forward to discovering more about the mechanism of the link between these two diseases, as well as new applications of GLP-1R agonists in the treatment of AD.

## 2 GLP-1R in AD

GLP-1R plays a key role in AD. The role of GLP-1R in AD is reflected mainly in the following aspects: it can decrease Aβ deposition and inhibit tau protein hyperphosphorylation, reduce neuroinflammation and oxidative stress (OS) (Du et al., 2022).

### 2.1 GLP-1R and Aβ deposition

**FIGURE 1:**
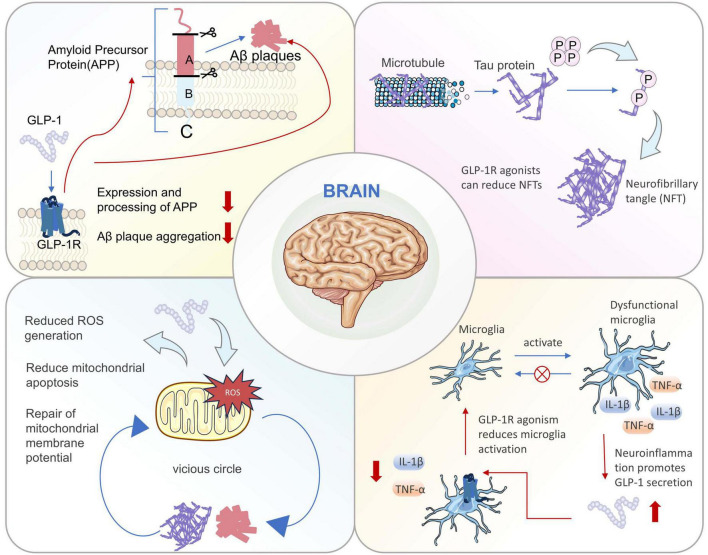
Simplified schematic of the four major pathological changes present in the brains of patients with AD and the role of GLP-1R. Multiple pathological changes, including Aβ deposition, tau protein hyperphosphorylation, neuroinflammation, and mitochondrial dysfunction, occur in the brains of patients with AD. GLP-1R activation plays significant modulatory roles in all these pathological mechanisms.

Aβ is a protein fragment produced by the cleavage of amyloid precursor protein (APP) by a series of enzymes and is one of the key factors in AD research (Hardy and Selkoe, 2002; Sambamurti et al., 2002; Figure 1). Two main forms of the Aβ protein have been identified: Aβ40 and Aβ42, which contain 40 and 42 amino acid residues respectively (Sambamurti et al., 2002). In AD, the Aβ protein exists as insoluble aggregates that can form larger plaques called amyloid plaques or senile plaques, which are deposited in the brain and interfere with the function of nerve cells (Roher et al., 1996). Abnormal accumulation of Aβ proteins is thought to be associated with neurodegenerative processes that may lead to disruption of communication between neurons, causing an inflammatory response and ultimately neuronal damage and death (Hardy and Allsop, 1991). Therefore, an imbalance in the production and clearance of the Aβ protein is a key part of AD pathology.

Several studies have confirmed that GLP-1R reduces Aβ production and deposition. GLP-1R agonists (e.g., exendin-4 and liraglutide) reduce APP expression and processing in the brains of AD model mice through the activation of GLP-1R, decrease Aβ protein production and plaque aggregation, and thus improve their spatial memory capacity (McClean et al., 2015; Wang et al., 2016). Studies have shown that liraglutide reduces the numbers of Aβ and dense core plaques in the cortex by 40–50% (McClean et al., 2011). In addition, defects in the insulin pathway lead to Aβ accumulation (Kellar and Craft, 2020). Jantrapirom et al. reported that liraglutide effectively reversed the deleterious effects of insulin overstimulation and attenuated neuronal insulin resistance in the human neuroblastoma cell line SH-SY5Y, which resulted in reductions in β-amyloid formation and tau hyperphosphorylation (Jantrapirom et al., 2020). GLP-1R activation also enhances the clearance of Aβ. GLP-1R is expressed predominantly at perivascular sites in astrocytes of the normal mouse cerebral cortex. Increased GLP-1R signaling promotes the phosphorylation and translocation of aquaporin 4, which may facilitate Aβ efflux clearance from the brain parenchyma by increasing intracerebral water flux (Sasaki et al., 2024). In summary, GLP-1R can influence the pathological process of Aβ in diverse ways, including Aβ production, deposition and degradation.

### 2.2 GLP-1R and tau protein hyperphosphorylation

Hyperphosphorylated tau is a major component of intracellular neurofibrillary tangles (NFTs), which, together with amyloid plaques, are a distinguishing marker of AD (Tracy et al., 2022; Figure 1). Normally, tau proteins exist in a microtubule-bound form, but in AD, tau proteins become hyperphosphorylated, forming NFTs (Samudra et al., 2023). These NFTs accumulate inside neurons and interfere with intracellular transport, leading to impaired cell function and neuronal death (Macdonald et al., 2019).

In recent years, studies on the use of GLP-1R and GLP-1R agonists to reduce tau protein hyperphosphorylation have progressed. Liraglutide and dulaglutide, as GLP-1R agonists, can improve AD-related cognitive dysfunction by inhibiting tau protein hyperphosphorylation and NFTs formation through activation of the protein kinase B/glycogen synthase kinase 3 beta (Akt/GSK-3β) signaling pathway (Shu et al., 2019; Zhou et al., 2019). This effect can be specifically blocked by the GLP-1R antagonist exendin (9–39) amide. Furthermore, exendin-4 can stimulate the cyclic adenosine monophosphate/protein kinase A (cAMP-PKA) pathway by activating GLP-1R, which then increases the level of non-phosphorylated β-catenin to stimulate the Wnt/β-catenin/NeuroD1 pathway and inhibits the activity of GSK-3β, ultimately decreasing the hyperphosphorylation of AD-associated tau proteins regulated by GSK-3β (Kang et al., 2023). In summary, GLP-1 agonists do not affect tau phosphatase activity but rather inhibit tau hyperphosphorylation by the activation of Akt-driven GSK-3β inhibition by GLP-1R during AD (Reich and Hölscher, 2022).

Although studies in animal models have shown that GLP-1R agonists reduce Aβaccumulation and tau hyperphosphorylation, few human studies have evaluated these effects. Clinical trials are still needed to validate their safety and efficacy in human patients before they can be widely used in AD therapy. And Clinical trials have been conducted to investigate the potential cognitive benefits of GLP-1R agonists in patients with AD. For example, the REWIND trial revealed that dulaglutide may reduce the risk of cognitive decline in patients with T2DM (Cukierman-Yaffe et al., 2020; Table 1). Novo Nordisk conducted a randomized, double-blind, placebo-controlled phase 2b clinical trial called ELAD to evaluate the neuroprotective effects of liraglutide in patients with mild AD (Femminella et al., 2019). Unfortunately, its primary endpoint was not met due to study limitations. In addition, two large-scale, double-blind, placebo-controlled phase 3 clinical studies called Evoke and Evoke + are underway to investigate the disease-mitigating potential of semaglutide in patients with AD with early symptoms and to explore its effects on AD biomarkers and neuroinflammation (Cummings et al., 2025). GLP-1R agonists show great potential in AD, but key challenges, such as blood brain barrier (BBB) penetration, clinical trial inconsistency, long-term safety and precision therapy, need to be addressed. In the future, multitargeted drugs, novel delivery technologies, and individualized treatments may propel them to become breakthrough therapies for a wider range of diseases.

**TABLE 1:** Summary of the mechanisms of action and effects of GLP-1R agonists in AD.

GLP-1R agonist	Model type	Mechanisms/methods of action	Primary effects	Ref.
**Exenatide/Exendin-4**	db/db mice, high-fat-diet/streptozotocin—induced diabetic mice, and high-glucose-damaged HT-22 hippocampal cells	Activates the Wnt/β-catenin signaling pathway and upregulates the expression of NeuroD1. Activates the insulin signaling pathway and inhibits GSK-3β activity.	Attenuates tau hyperphosphorylation and cognitive dysfunction, ameliorates learning and memory deficits, and exerts its protective effects by increasing brain-derived insulin levels.	Kang et al., 2023
	Clinical research	Reduces levels of pro-inflammatory cytokines, decreases expression of vascular cell adhesion molecules, and modulates immune system functions.	Significant inflammatory protein levels correlate, perhaps aiding in the progression of AD symptoms by modulating chronic inflammation.	Koychev et al., 2024
**Liraglutide**	Human neuroblastoma cell line SH-SY5Y	Improvement of neuronal insulin signaling, reversal of the phosphorylation state of the insulin receptor and its downstream signaling molecules (e.g., IRS-1, AKT, GSK-3β), and inhibition of β secretase 1 or β-site APP-cleaving enzyme 1 activity.	Reduces tau hyperphosphorylation and Aβ deposition and restores insulin sensitivity in neurons damaged under hyperinsulinemic conditions.	Jantrapirom et al., 2020
	Transgenic hTauP301L mice	Activation of GLP-1R inhibits upstream signaling pathways associated with tau phosphorylation (e.g., GSK-3β activity).	Significantly reduces phosphorylated tau load in midbrain and hindbrain-associated neurons and improves neurological function in hTauP301L transgenic mice.	Hansen et al., 2016
	Clinical research	Changes the brain glucose metabolic rate.	Significant differences in cognitive function were not reached (probably due to insufficient sample size).	Femminella et al., 2019
**Dulaglutide**	*In vitro* cell modeling	Activates microglia and promotes their polarization toward an anti-inflammatory phenotype (type M2).	Ameliorates Aβ-induced inflammation and neuronal injury and mediates microglia activation and polarization.	Wang and Han, 2025
	C57/BL6 male mice	Modulation of the PI3K/AKT/GSK3β signaling pathway reduces hyperphosphorylation of tau protein and neurofilaments.	Significantly improves learning and memory deficits in STZ-induced AD-like mice.	Zhou et al., 2019
	Clinical research	An exploratory analytical approach through an international multicenter, randomized, double-blind, placebo-controlled trial (REWIND trial).	Dulaglutide may reduce cognitive impairment in people aged 50 years or older with T2DM.	Cukierman-Yaffe et al., 2020
**Semaglutide**	Human neuroblastoma cell line SH-SY5Y	Preventing 6-OHDA cytotoxicity in SH-SY5Y cells by improving autophagic flux, reducing oxidative stress and attenuating mitochondrial dysfunction.	Shows neuroprotective effects.	Liu et al., 2022
	APP/PS1/Tau transgenic mice	Enhancement of glucose uptake and utilization in the brain through SIRT1 activation of GLUT4 expression and transport.	Significantly improves cognitive function, reduces the burden of Aβ and tau pathology, and enhances glucose metabolism in the brain.	Wang Z. J. et al., 2023
	Clinical research	Ongoing AD trials.	The EVOKE trial: assessing the impact on cognitive function in patients with early AD.	Cummings et al., 2025
**Tirzepatide**	Human neuroblastoma cell line SH-SY5Y	Activates the pAkt/CREB/BDNF signaling pathway and its downstream cascade reactions to affect DNA methylation and miRNA expression.	Ameliorates high glucose-induced neurodegeneration and overcomes neuronal insulin resistance.	Fontanella et al., 2024
	APP/PS1 mouse model	Activates GLP-1R, regulates brain glucose metabolism, enhances the expression of glucose transport and metabolism-related genes, and ameliorates mitochondrial dysfunction.	Significantly reduces the number of Aβ plaques, decreases neuronal apoptosis, and ameliorates mitochondrial dysfunction in astrocytes in the brains of APP/PS1 mice but does not significantly affect anxiety or cognitive function in the mice.	Yang et al., 2024
**Lixisenatide**	APP/PS1/Tau AD transgenic mice	Activates the PKA–CREB signaling pathway and inhibits the p38–MAPK signaling pathway to exert its neuroprotective effects.	Significantly reduces Aβ plaques, NFTs, and neuroinflammation in the hippocampal region of mice.	Cai et al., 2018

### 2.3 Others

In addition to Aβ deposition and tau protein hyper- phosphorylation, pathological changes such as neuroinflammation and mitochondrial dysfunction are closely related to AD pathogenesis. Research indicates that the activation of GLP-1R can alter the polarization of microglia, shifting them from a proinflammatory (M1) phenotype to an anti-inflammatory (M2) phenotype (Qian et al., 2022). This shift is crucial because the proinflammatory cytokines released by M1 microglia can exacerbate neuronal damage, whereas M2 microglia exert protective effects by promoting anti-inflammatory responses and repair processes (Jassam et al., 2017). Therefore, GLP-1R is expressed in glial cells and has anti-inflammatory properties (Calsolaro and Edison, 2016). The activation of GLP-1R using agonists such as exendin-4 has been reported to reduce microglial activation and the production of proinflammatory cytokines (Qian et al., 2022). This effect contributes to the protection of neuronal tissue and the improvement of functional recovery after injury. OS is another key factor in AD, which leads to synaptic damage, and GLP-1R is able to reduce OS and protect synaptic structure and function (Kong et al., 2023; Liang et al., 2024). GLP-1R deletion impairs mitochondrial integrity in astrocytes, and a lack of GLP-1R signaling impairs mitochondrial function and induces a cellular stress response (Timper et al., 2020). Recently, GLP-1 was shown to act on GLP-1R and exert its neuroprotective effects by promoting PTEN-induced kinase 1/Parkin-mediated mitochondrial autophagy and attenuating OS (Wang Y. et al., 2023). These findings further confirm the pleiotropic role of GLP-1R in neuroprotection.

## 3 GLP-1R as a link between DM and AD

**FIGURE 2:**
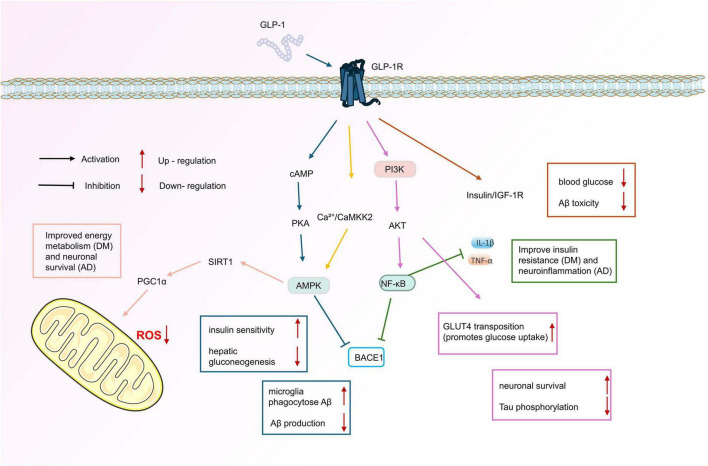
Schematic representation of signaling pathways common to AD and DM induced by GLP-1R agonism. These include AMPK, PI3K/AKT, CaMKK2-AMPK, NF-κB, insulin/IGF-1 R and the mitochondrial signaling pathway.

Our previous chapter discussed the role of GLP-1R in AD. Why is it that GLP-1R could be a potential link between DM and AD? The mechanisms of action of GLP-1R in DM and AD overlap, which suggests that GLP-1R may be a bridge between these two diseases. For example, GLP-1R agonists were able to improve glucose metabolism function through the GLP-1R/SIRT1/GLUT4 pathway in an AD model (Wang Z. J. et al., 2023), suggesting that GLP-1R may influence AD progression by modulating metabolic pathways associated with DM. In addition, GLP-1 activation in astrocytes by GLP-1R altered cellular glucose metabolism, revealing a novel mechanism by which GLP-1R improves cognitive function in patients with AD (Zheng et al., 2021). Taken together, these findings highlight the common mechanism of action of GLP-1R in DM and AD and its potential to regulate metabolism and neuroprotection (Figure 2), suggesting that GLP-1R may be a key factor linking these two diseases.

### 3.1 Brain insulin resistance

Insulin resistance in the brain perpetuates neuroinflammation, tau hyperphosphorylation, and amyloid pathology in AD and is therefore a driver of neurodegenerative disease (Hölscher, 2019). This has led some researchers to refer to AD as “type 3 diabetes” because of the similarities between the impaired brain insulin signaling observed in AD and the insulin resistance observed in T2DM (see below) (de la Monte, 2014; Steen et al., 2005). However, this nomenclature has been controversial, with some scholars arguing that categorizing AD as “type 3 diabetes” may be conceptually misleading (Talbot and Wang, 2014; Li et al., 2024). The traditional classification of DM is mainly based on abnormalities in insulin secretion and action, such as type 1 DM due to an absolute lack of insulin secretion and T2DM due to insulin resistance and relative insulin deficiency. However, the inclusion of abnormal insulin metabolism in the brain as part of “type 3 diabetes” is a break from conventional wisdom and therefore has not yet been agreed upon in the academic community. Firstly, some people believe that cerebral insulin resistance in patients with AD may not be insulin resistance in the true sense of the word but may instead arise from dysfunctional insulin transport across the BBB and that this transport defect may be caused by abnormal BBB function indirectly resulting from peripheral insulin resistance (Arnold et al., 2018). Second, existing animal models have significant limitations. Although rodent models provide important tools for AD research, it is difficult for these models to fully simulate the complex pathophysiological processes of human AD because of the significant differences in brain structure, metabolic characteristics, and immune responses between humans and experimental animals (Qian et al., 2024). However, abnormal desensitization of insulin signaling has been observed in the brain tissue of patients with AD even in the absence of DM (Frölich et al., 1998).

Multiple parallels between impaired brain insulin signaling in AD and insulin resistance in T2DM have been reported. Insulin and insulin-like growth factor-1 (IGF-1) play important roles in cognitive performance, neurological function, and the control of neurogenesis and synaptogenesis (Choi et al., 2025). Insulin-degrading enzymes (IDEs) are enzymes used to break down insulin and IGF-1, removing Aβ_40_ and Aβ_42_ monomers but not affecting Aβ oligomers or fibers (Kemeh and Lazo, 2023). In an insulin-resistant milieu, insulin may competitively inhibit IDE, which impedes the degradation of Aβ proteins, increases their neurotoxicity, and contributes to the onset of AD (Scherer et al., 2021; Ochiai et al., 2021). In the state of brain insulin resistance, insulin signaling pathways such as the PI3K/Akt pathway become abnormal, and abnormalities in insulin signaling pathways lead to a decrease in Aβ clearance, which promotes Aβ deposition (Zheng and Wang, 2021). In addition, brain insulin resistance affects tau metabolism and promotes the hyperphosphorylation of tau proteins, tau protein aggregation, the formation of paired helical filaments, and the further formation of NFTs (Mohandas et al., 2009). GLP-1, as an insulin-promoting hormone, has functional and growth factor properties similar to those of insulin and IGF-1 (Bhalla et al., 2022). GLP-1R, as its receptor, can bind to GLP-1 to exert its growth factor effects. In addition, Aβ has a tertiary structure similar to that of insulin, peripheral Aβ acts as a negative regulator of insulin secretion, and there can be interactions between Aβ and insulin signaling (You et al., 2022).

Brain insulin resistance is an important pathogenetic feature of AD and is mediated primarily by impaired insulin signaling (Sêdzikowska and Szablewski, 2021). In a study using the GLP-1R agonist liraglutide, its ability to reverse cognitive deficits in an AD model and its potential neuroprotective mechanisms were identified. Liraglutide not only blocks insulin receptor and synaptic loss in the brain but also reverses memory impairment induced by AD-associated Aβ oligomers, suggesting that GLP-1R activation may be used to protect brain insulin receptors and synapses in AD (Batista et al., 2018). In addition, GLP-1R stimulation activates insulin signaling pathways and regulates gene expression, decreasing systemic insulin resistance and brain insulin resistance in patients with AD (Dahiya et al., 2025). Moreover, because GLP-1R is expressed throughout the body, stimulation with a GLP-1R agonist or indirectly with a DPP-IV inhibitor can have a broad systemic effect on systemic metabolism, which, in turn, ameliorates peripheral and central insulin resistance in AD and MD (Athauda and Foltynie, 2016). Therefore, it is reasonable to believe that GLP-1R is a potential link between these two diseases.

### 3.2 Neuroinflammation

AD and DM are significantly associated with neuroinflammatory mechanisms. Chronic low-grade inflammation is a common pathological feature of both: metabolic disturbances in patients with DM induce the release of peripheral inflammatory factors, and these inflammatory factors pass through the compromised BBB into the central nervous system (CNS), activating microglia and astrocytes and triggering a neuroinflammatory cascade response, which in turn promotes Aβ deposition in AD and tau protein hyperphosphorylation (Sebastian Monasor et al., 2020; Chen et al., 2024). In addition, obesity-associated adipose tissue inflammation further exacerbates CNS inflammation, creating a vicious cycle of “metabolism–inflammation–neurodegeneration” (Chen et al., 2024; Wong et al., 2024).

GLP-1R plays a multidimensional role in regulating neuroinflammation. Firstly, through a systematic review and network meta-analysis, researchers have assessed the effects of GLP-1R agonists on neuroinflammation and reported that, compared with placebo, GLP-1R agonists significantly reduce the levels of neuroinflammatory markers, such as TNF-α and interleukin-1β (Urkon et al., 2025; Zhang et al., 2022; Tseng et al., 2025). Second, GLP-1R activation enhances neurovascular coupling function, improves cerebral blood flow and repairs BBB integrity, blocking the penetration of peripheral inflammatory factors into the center (Wong et al., 2024). Preclinical studies have also revealed that dual agonists of GLP-1R and glucose-dependent insulinotropic polypeptide receptor (GIPR) have synergistic anti-inflammatory and neuroprotective effects, suggesting the potential advantages of multitargeting strategies (Yuan et al., 2024). GLP-1R-targeted therapies have now expanded from metabolic diseases to AD. A team of researchers developed a nanostructure-based GLP-1R agonist capable of crossing the BBB that significantly attenuated neuroinflammation and memory loss in an Aβ peptide-induced mouse model of AD by inhibiting the inflammatory responses of microglia and astrocytes (Zhao et al., 2022). These findings not only reveal the potential of GLP-1R as a common therapeutic target for AD and DM but also provide a theoretical basis for the development of novel therapies based on the “metabolic–immune–neurological” axis.

### 3.3 Mitochondrial dysfunction and oxidative stress

Chronic hyperglycemia in patients with T2DM leads to peripheral insulin resistance, whereas impaired insulin signaling pathways in the brains of patients with AD lead to “brain insulin resistance,” both of which are closely related to mitochondrial dysfunction and OS (Du et al., 2022; Zhang et al., 2023). Mitochondria are the primary site of energy metabolism and reactive oxygen species (ROS) production. Hyperglycemia exacerbates mitochondrial electron transport chain (ETC) dysfunction and increases ROS production through advanced glycation end products and inflammatory pathways (Zhang et al., 2023; Caturano et al., 2023). Overproduction of ROS triggers OS (Luna-Marco et al., 2023). Interestingly, defects in mitochondrial energy metabolism are also present in the brains of patients with AD, leading to neuronal apoptosis and Aβ deposition, which increases ROS production by interfering with mitochondrial calcium homeostasis and ETC function; the hyperphosphorylation of tau proteins leads to the disruption of microtubule structure and affects mitochondrial axonal transport, exacerbating the neuronal energy crisis (Meng et al., 2024). Activated microglia in patients with AD release proinflammatory factors, which further promote ROS production, creating a vicious cycle of neuroinflammation and mitochondrial dysfunction (Qian et al., 2025).

GLP-1R can restore the mitochondrial membrane potential, promote ATP production, and reduce ROS production by activating the cAMP/PKA pathway (Signorile et al., 2022). In addition, activation of GLP-1R regulates mitochondrial over fission by the cAMP/PKA pathway while improving mitochondrial function in Aβ-treated astrocytes and ameliorating pathological lesions in AD (Xie et al., 2021). Clinical studies have shown that GLP-1R reduces ROS levels, the mitochondrial membrane potential and mitochondrial apoptosis in patients with diabetes (Durak and Turan, 2023; Wang et al., 2021), as well as alleviating levels of OS and attenuating low-grade inflammation (Zhang et al., 2018). GLP-1R reduces oxidative damage accumulation by modulating autophagy-related proteins and scavenging damaged mitochondria while increasing superoxide dismutase (SOD) and glutathione peroxidase activities, reducing the generation of lipid peroxidation products, inhibiting the NF-κB signaling pathway, and decreasing proinflammatory factor expression to reduce neuroinflammation (Ma et al., 2018; Lin et al., 2021). In conclusion, GLP-1R plays important roles in mitochondrial dysfunction and OS in AD and DM.

## 4 Conclusion

The treatment of AD faces serious challenges, and the incidence of this disease is increasing every year, placing a heavy burden on global health. Despite the never-ending exploration of AD, our understanding of the disease remains limited, especially in terms of etiology and pathogenesis. Recent studies suggest that GLP-1R may be an important link between DM and AD. Evidence suggests that GLP-1R agonists, initially developed for the treatment of DM, have therapeutic potential in the management of AD because of their multifaceted mechanism of action. GLP-1R agonists exhibit neuroprotective effects in AD, including anti-inflammatory effects, modulation of Aβ deposition and clearance, improved insulin signaling, and attenuation of OS. The intersection between DM and AD further highlights the shared pathophysiological mechanisms, particularly the disruption of insulin signaling pathways in the brain. This disruption is referred to as “type 3 diabetes” and is characterized by neuroinflammation, cognitive deficits, and amyloid pathology, which are common to both DM and AD. GLP-1R may ameliorate these conditions by improving insulin signaling and reducing insulin resistance in the brain.

Although GLP-1R agonists have yielded promising results in animal models, AD transgenic mice do not fully mimic the complex pathology of human AD, and there are still some challenges in translating them into effective AD therapies. For example, limitations in BBB penetration efficiency allow for limited distribution in the CNS, which may affect efficacy. Current studies suggest that peripherally administered GLP-1RA has low concentrations in the cerebrospinal fluid, and higher doses or improved delivery systems (e.g., nanoparticles, liposome encapsulation) may be needed to increase brain exposure. In addition, there are potential risks and limitations associated with GLP-1R therapy. Most GLP-1R agonists have gastrointestinal side effects, including nausea, vomiting, diarrhea and constipation, which may be more pronounced in elderly patients with AD and affect treatment compliance. Whether long-term use leads to risks such as hypoglycemia and thyroid C-cell hyperplasia remains to be further evaluated.

However, novel drug delivery systems or formulations may be able to reduce the risk of gastrointestinal side effects and hypoglycemia. With the in-depth theory of the gut–brain GLP-1R axis, the breakthrough of new material technology and the rapid development of AI-assisted drug design, GLP-1R-related research has also ushered in new opportunities. An in-depth analysis of the signaling mechanism of GLP-1R in the gut–brain GLP-1R axis is needed to aid in developing smarter new material delivery systems to achieve precise targeting and long-lasting release of GLP-1R agonists. Moreover, AI technology can be used to accelerate the design and screening of novel GLP-1R agonists to promote personalized therapy. In conclusion, GLP-1R signaling represents a promising therapeutic strategy that bridges the treatment of DM and AD. Its potential to modulate metabolic and neuroprotective pathways offers hope for the development of new therapies that could improve the prognosis of patients with both diseases. GLP-1R is not only a key target for metabolic regulation but also a bridge between metabolism and the nervous system. With further research and technological advances, GLP-1R agonists are expected to become the core drugs for the treatment of AD and DM. Future studies should continue to explore the dual mechanism of action of GLP-1R in metabolism and the nervous system, especially its potential applications at the intersection of AD and DM.
